# Polymers, pastries, and penguins: An interview with Peter Barham, professor of physics, molecular gastronomy and conservation biology

**DOI:** 10.1038/s44319-023-00025-1

**Published:** 2024-01-08

**Authors:** Adam Gristwood

**Affiliations:** Hadfield, UK

**Keywords:** Economics, Law & Politics, Evolution & Ecology, History & Philosophy of Science

## Abstract

An interview with Peter Barham about science, cooking and penguins.

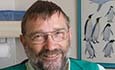

**Figure:**
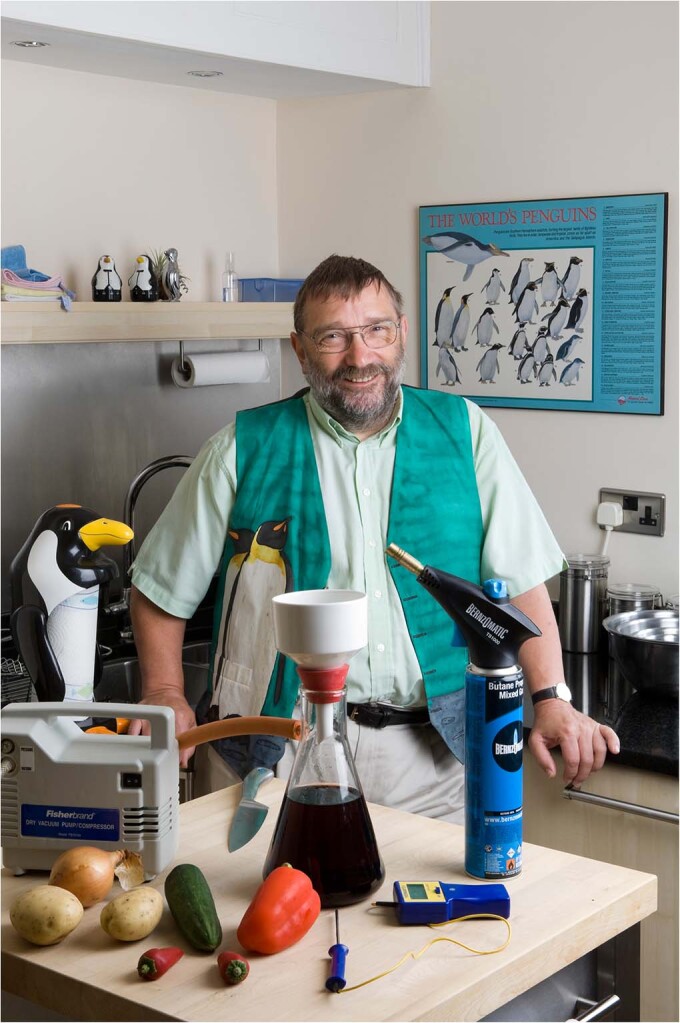
Peter Barham gained his PhD in Polymer Physics from Bristol University in 1975 and has worked in the physics department ever since, first as a post-doc researcher and later as a lecturer and a professor. In addition to several honorary posts in polymer physics at various universities around the world, he has also held positions at the University of Copenhagen (Professor of Molecular Gastronomy), and in South Africa at the University of Cape Town where he was an honorary research associate in Biological Sciences and at the University of the Western Cape where he is now an Extraordinary Professor of Conservation Biology. During his career, Peter has managed to combine his love of Physics with his two other passions, cooking and penguins. He has advised and assisted several chefs to introduce scientific methods and techniques into their kitchens, most notably Heston Blumenthal and has become very active in promoting the conservation of African penguins. Image: Dave Pratt/University of Bristol with permission.

**EMBO Reports (ER):** Your roles have included Professor of Physics, honorary Professor of Molecular Gastronomy and Extraordinary Professor of Conservation Biology: even in today’s multidisciplinary science landscape this is an unusual spread of skills. Could you reflect on the key moments that inspired you into science in the first place?

**Peter Barham (PB):** As a child, I was always trying to understand how things work. Much to the dismay of my mother, I removed an electric motor from a toy train set and took it to pieces to better understand the mechanisms going on inside and make it go faster. She was stunned when I successfully reassembled it, while I thought to myself: I can do this! Years later, I was inspired by excellent teachers to study physics, and during my master’s, I was assigned a project on the polymer physics of how long-chain molecules crystallise. Not many people were interested in this at the time, however, I found it fascinating how during the crystallisation process, the arrangement of polymers is transformed from what looks much like a ball of tangled wool to highly regular crystalline solids.

There also appeared to be a lot of potential practical applications with polymer physics, especially in terms of making things stronger. I secured sponsorship for my PhD from a large UK-based chemical company and would occasionally visit the organisation’s headquarters to update them on my progress. After one visit, a colleague and I were challenged to find a way to control the heat seal strength of a polypropylene sheet—or in other words, how to control the sealing on a packet of crisps. Snack makers had long had problems with the seal on packaging: do it firmly, and the polymer melted, do it lightly, and the pack leaked air and the product quickly went stale. In the lab, we were able to make a method work and were quickly invited back to give a demonstration, with the company delighted by the results. There were rumours that the technology we developed allowed the firm to manipulate the seal to ensure that some packets spilled contents—and forcing people back to buy a new packet. Nevertheless, the project’s success was a revelation to me: I realised that by understanding more about how polymers melted and crystallised, we could potentially solve a lot of everyday problems.

**ER:** What did you find the hardest?

**PB:** I was invited to work on a project involving a brand-new material called polyhydroxybutyrate (PHB), a biodegradable polymer produced by bacteria. It’s fantastic stuff: the only naturally occurring thermoplastic we know of, and it can be used in all the same ways as polyethylene, polypropylene, and nylon. I was motivated by the potential to develop environmentally friendly plastic alternatives. The difficulties of processing PHB into flexible, thin films on a large scale has been one of the reasons restricting its widespread applications. However, another factor that has reduced concerted efforts to overcome these problems is political.

During the oil crises in the 1970s, there was an urgency to explore ways of producing plastics without using oil. However, as the price of oil fell, it became much cheaper to make Polyvinyl Chloride (PVC) and the incentives to push forward the technology fell by the wayside. On the other hand, there has long been interest in academia to develop approaches to plastic that reduce impacts on the environment. For methods such as pyrolysis to really have a global impact would require fundamental changes to the plastics and oil industries—including the phasing out of PVC which contain chlorinated polymers, that would wreck the system.

Vested interests have long blocked efforts to secure these changes or led to approaches that have done more harm than good such as adding chlorine from seawater into plastics, which turned out to be massively polluting when burned. When we tried to raise the alarm, we experienced a lot of resistance, as the industry talked it up as ‘environmentally friendly solutions’ but in reality it was a pure public relations exercise, and they clearly did not give a damn. These experiences, while very frustrating, have also been motivating in a way and over time have inspired me to become more involved with science communication and science-based policy advice.

When we tried to raise the alarm, we experienced a lot of resistance, as the industry talked it up as ‘environmentally friendly solutions’ but in reality it was a pure public relations exercise…

**ER:** What led you into food science?

**PB:** When I finished my PhD, I went from working day and night on my project to suddenly having a whole load of free time on my hands. My wife bought me a cookbook and suggested that I try baking. I picked out a delicious-looking cake, and was very confident at first: as a properly trained scientist I should very well know how to measure ingredients and follow instructions. However, despite being sure that I had followed the recipe to the letter, what came out of the oven was a disaster, and looked more like a badly made pancake. I had a few more tries, achieved the same results, and realised there must be a problem with the instructions: no matter how many times I did it, a cake would never rise. So I went back to the basics and tried to work out what the instructions should have been. Eventually, a half-decent cake came out of the oven and solving this seemingly straightforward problem using first principles showed me that there is more to life than polymer physics.

… despite being sure that I had followed the recipe to the letter, what came out of the oven was a disaster, and looked more like a badly made pancake.

Nevertheless, I persisted in the field and during my postdoc it was a team tradition to bring cakes in on one’s birthday—and rather than going to the bakery I decided to make mine myself. People enjoyed them so much that I had regular requests to bake on behalf of others. After a year or so of fulfilling orders in my spare time I had quite enough and decided that I would teach people how to make cakes for themselves. I managed to secure a slot on our department’s research seminar programme, disguised under the title ‘Structurally Reinforced Composite Foams From Natural Materials’. This was essentially a live demonstration of how to make a Black Forest gateaux, explaining the physics and chemistry of why the process worked, and where it could have gone wrong. A colleague happened to be in the audience who organised public lectures and invited me to join their programme. One thing led to another and suddenly I was giving regular public talks and working with all manner of organisations on how to bring science into their kitchens, from the Women’s Institute to Michelin-starred restaurants.

**ER:** What brought you from the lab into the kitchen?

**PB:** My boss Andrew Keller was contacted by his friend Nicholas Kurti, an expert on low-temperature physics who was very enthusiastic about applying science in the kitchen. Kurti was co-organising a workshop on food and science in Erice, Sicily, and was looking for natural scientists to join chefs and food experts who explored what happened in the kitchen—and why it worked or didn’t work. I imagine the conversation went something like: ‘we have this kid working with us, he does some stuff with food, he is a little bit crazy but what the heck—he can join!’. So in 1992, I was sent along to the meeting and really enjoyed the experience: there were Michelin-starred chefs, Nobel Prize-winning scientists, and a whole range of other experts together in the same room.

The Erice events were largely credited for coining the term ‘molecular gastronomy’, however what I really took away from them was how well cooks and scientists could work together to solve problems. I continued to give public lectures, and as my public profile grew I began to learn the importance of understanding how the media works. This paid off: some years later, I was sitting in my office and received a phone call from Heston Blumenthal, now regarded as a pioneer of multi-sensory cooking and food pairing. Blumenthal wanted to know if I had the answer to a basic cooking question: should one put salt in the water when cooking green beans? I told him that it was utterly pointless: there is no good reason to. He was pleased with the reply because the kitchen in his restaurant, the triple Michelin star Fat Duck, was small and cutting out this method saves time and space.

The Erice events were largely credited for coining the term ‘molecular gastronomy’, however what I really took away from them was how well cooks and scientists could work together to solve problems.

If you ask ‘why?’ and the answer is ‘because you have to’, it’s important to question if that’s really true, especially if you have evidence to the contrary. In the past, unrefined salt was added to water to make vegetables that were browning look green again. This happens when magnesium or calcium is dissolved in the water at boiling temperatures. However, nowadays, we use refined salt that contains none of these minerals, so there is no point in adding this to the water. I have found that there are so many things in recipes that have little point: another is instructions to sieve flour, which used to be necessary to get the weevils out, but now flour comes pre-sieved. I have spent a lot of time convincing senior chefs with demonstrations and blind taste tests and working with them to integrate scientific approaches into their work.

If you ask ‘why?’ and the answer is ‘because you have to’, it’s important to question if that’s really true, especially if you have evidence to the contrary.

Blumenthal was the first chef I came across who was able to put the science back into the kitchen: he was not classically trained, didn’t have any baggage, and was very excited about what we could do together. Every other Monday, he would drive from London to Bristol and we would work together in the lab: we had temperature control, distillation and vacuum kits, liquid nitrogen, and we played around with different ideas and observed what happened. Tools we developed that worked well often ended up in his restaurant: temperature-controlled water baths to cook fish and meat, vacuums that could extract flavours from herbs and stock before they were lost to the environment, several efforts that broke world ice-cream-making records.

Heston Blumenthal was the first chef I came across who was able to put the science back into the kitchen: he was not classically trained, did not have any baggage, and was very excited about what we could do together.

Much of the focus was on a scientific understanding of flavour and taste: how the eating experience is affected by colour, noise and texture. We collaborated like I would with any other scientist, just sometimes our conversations might quickly veer off in unexpected directions. Chefs saw what Blumenthal was doing and started taking this scientific approach to cooking seriously. I like to think of it more as a set of tools: There was nothing magical about it, it’s rather having a scientific understanding and knowing what to do.

**ER:** What ideas did you have that didn’t work out so well?

**PB**: One thing that we kept trying and failing to create was levitating food. These efforts culminated in a floating dessert in which a meringue rests on a pillow that ‘floats’ and spins around 15 cm above the table. But rather than genuinely hovering, an illusion is created through projection, optics and a concave mirror. Other things worked well in the lab, but not so well in the restaurant. One example is our attempts to create a dish that heats up as you eat it. When you have a liquid and you turn it into a solid, it gives off heat as it crystallises: We created a superheated liquid that solidifies and gives off heat as it cools down in the mouth, but when it came to testing it in the restaurant we could only get it to work about half the time. We had so many ideas, that Heston would keep asking: can we make it work? Sometimes I would answer yes, other times no, and sometimes years later someone else would come along with a method we did not think of. While our experiments inevitably led to a lot of disasters in the lab, none that I know of were ever served to diners at their table.

**ER:** What can scientists learn from chefs?

**PB:** In many respects, the best chefs are very scientific in their approaches because they keep trying and trying until they get a result. The main difference is that in science we often do not know what to expect from the results, while in a restaurant, one has a vision of what it should look like, taste like, its texture, and keeps going until the vision is achieved. This requires a lot of trial, error, and intuition, and there is a learning curve that comes from this approach that I think is valuable for any specialist carrying out any type of scientific work. Chefs and scientists can be tremendous collaborators because both sides get something from the other in the process. A lot has changed in the past two decades, and most cookery places have now got science in their curriculum, so I have taken a step back: there are plenty of younger experts coming into the field with new ideas and approaches and my work is now largely focused on penguin conservation.

In many respects, the best chefs are very scientific in their approaches because they keep trying and trying until they get a result.

**ER:** That is another remarkable career change: how did that happen?

**PB:** There are some surprising similarities to previous roles, not least because my involvement is again largely down to my wife. She has always been a big fan of penguins, and in the mid-1990s we saved every penny we could to take a trip to Antarctica to see them in the wild. It was an amazing experience and, keen to learn more, we attended an international penguin conference in South Africa. One of the speakers was the late Bernard Stonehouse, a renowned expert in penguin behaviour. Stonehouse had long worried about ill-effects caused by the metal bands that researchers used to monitor their movement. Exasperated by what he saw as inertia in the community in addressing the problem, he gave an impassioned talk about the need to find a better way.

One of the ideas discussed during the session involved the use of plastic bands and the discussion became quite heated, with people in the room with no specific knowledge on the subject debating the pros and cons. At this point, my wife nudged me into saying something: I introduced myself as an amateur penguin enthusiast who happened to know a thing or two about plastics and before I knew it, I had been volunteered to make something penguin- and environmentally friendly. Back in Bristol, we began undergraduate projects to see if we could come up with a solution, tested them on penguins in a local zoo, and then secured funding to trial the bands in the wild in studies of the African penguin.

The trial was due to begin in 2001, however, it nearly did not happen because a disastrous oil spill occurred around 10 km off the coast of South Africa, caused by the sinking of the *MV Treasure* cargo ship. The spill affected around 40,000 penguins, with rescuers focussing on three main strategies to try and minimise damage—to clean up oiled birds, relocate affected birds, and to hand-rear chicks who had been orphaned by the catastrophe. Most of these birds were fitted with the old metal bands which made it hard for us to find birds that were not already banded to use in our project. However, we did persist and managed to compare the breeding success of birds fitted with different types of bands. So suddenly, we had all this data coming in that could allow us to track what happened to the birds that had been rescued as well as test novel types of band.

… suddenly we had all this data coming in that could allow us to track what happened to the birds that had been rescued as well as test novel types of band.

Some 5000 chicks were hand-reared by specialists and one of the striking things we found by analysing the observations was that contrary to perceived conventional wisdom, this strategy was the most successful. There had previously been an assumption that efforts to hand-rear chicks were largely in vain because they had not been reared properly and would not have the experience needed to survive in the wild. The data told us that these penguins in fact thrived in the wild, and this finding has changed the way that ecologists look at disasters: all three of the main strategies are important for minimising damage to colonies and populations. This catapulted me from being penguin enthusiast to an established penguin biologist and conservationist.

**ER:** What do you see as the main challenges in securing the long-term survival of the African penguin?

**PB:** At the moment, the situation is very worrying: at the turn of the millennium 40,000 African penguins were affected by the *MV Treasure* oil spill, but today there are not even 40,000 birds left in the wild in total. This has fallen from more than a million birds and continues to fall every year. Thus there are real concerns that the species will become functionally extinct in the wild in the next few decades.

There are a number of reasons for this decline, however, there appears to be strong scientific evidence that the main cause is overfishing and a lack of fish stocks. This has immersed me back in difficult political discussions, this time framed around fisheries policy. People in South Africa are very passionate about protecting the African penguin, however, unless something is done immediately to prevent its decline, then it will be too late.

The fishing industry is a major contributor to the South African economy […], so it’s not possible to just say ‘stop fishing’ and be done with it, and we have been working to try and develop a proper plan.

The fishing industry is a major contributor to the South African economy and there is still a lot of poverty and unemployment, so it’s not possible to just say ‘stop fishing’ and be done with it, and we have been working to try and develop a proper plan. For the past year this has involved the preparation of papers and arguments to look at all aspects of fisheries and fisheries policy as applied to penguins. It’s been a lot of work to keep this moving: like with my experiences with the plastics industry, we are coming up against organisations with serious money and influence. It feels a bit like a David versus Goliath problem, but if we do not aim to solve the issue, we won’t achieve anything, so we have to try.

**ER:** What are the main scientific goals now?

**PB:** We want to better determine key parameters such as the numbers of penguins, how these numbers are changing, how productive the birds are in breeding, how they move between colonies, how long they live, when and where they die, how many birds choose not to breed in a given year, and why. And we want to do all of this with minimum interference by continuing to develop non-invasive technologies.

There are so many questions we don’t know the answer to, however, I believe that one of the biggest issues we have to contend with is that a lot of mature adults cannot find enough food and energy to support them rearing chicks. Rather than breeding once a year, we see they are only doing so every five or six years. This is far below levels needed to keep populations stable and is completely unsustainable in the long run.

Teams working on collecting these data have developed some really good methods: a large proportion of the African penguin population is tagged with transponders. We have got readers set up at most colonies that have delivered more than 300,000 readings to date. We are trying to see if we can use that data rather than visiting the nests and disturbing the penguins.

When penguin breeding pairs nest, they typically lay one or two eggs, and one of the birds incubates those eggs, while the other one goes to sea and eats some food, and then comes back maybe a few days later, swaps, and then a few days later they swap again. After around 40 days, the chick hatches, at which point the birds also have to feed it and therefore come and go more regularly. When the chick gets big enough to leave, both penguins can go out to sea at the same time, so their behaviour is heavily dependent on whether they have chicks and how old they are.

By installing readers on the ground, you can see when birds go and come back thanks to a signal sent by a transponder on the tags to the instrument. From the movements recorded, it’s possible to deduce whether the bird has got an egg, a small chick, or a big chick. We can then work out reasonably well, we think, how many breeding attempts are successful, how many fail, and calculate the breeding success for the colony.

There is a lot of high-quality, collaborative work going on between conservation organisations, research groups, the private sector, NGOs, and end users in developing methods, which we hope will also be a model for other conservation initiatives. Now the challenge is to get more into the nitty-gritty of the data: to do this, we have been exploring artificial intelligence and computer science approaches as well as looking for inspiration from other projects.

I spend a lot of my life now on Robben Island, getting my hands dirty, picking up penguins, getting bitten: I cannot think of anything else I would like to be doing more.

Another challenge comes back to communication: while a lot of fish are caught for other purposes, we actively engage with food retailers who in some respects are the public face of what happens to fish taken out of the ocean. So it’s looking at doing those sorts of campaigning things, which can hopefully lead to more sustainable solutions overall.

I have found that I have never really gotten what I expect—when you have one thing in mind, you do end up with something completely different.

**ER**: What’s next for you personally?

**PB**: I spend a lot of my life now on Robben Island, getting my hands dirty, picking up penguins, getting bitten: I cannot think of anything else I would like to be doing more. As I am retired, I have an advantage in that I do not have a job to lose: I can speak out and not have to worry so much about the consequences. No matter how strong the scientific arguments are to do something, they are being pulled by economic arguments in a totally opposite direction. But we are at the point where these economic arguments carry no weight: if we carry on business as usual, in the long-term there is a strong chance that fish stocks in parts of the Southern Atlantic will collapse. So part is on trying to show the importance of demonstrating long-term thinking and the benefits it could have for all. Another is to continue to take life as it comes: I have found that I have never really gotten what I expect—when you have one thing in mind, you do end up with something completely different. That’s what I love about being a scientist: it’s great.

**ER**: Professor Barham, many thanks for the interview.

The interview was conducted by Adam Gristwood.

